# Monogenec Arrhythmic Syndromes: From Molecular and Genetic Aspects to Bedside

**Published:** 2016

**Authors:** Golukhova E.Z., Gromova O.I., Shomahov R.A., Bulaeva N.I., Bockeria L.A.

**Affiliations:** Bakoulev Centre for Cardiovascular Surgery, Rublevskoye sh. 135, 121552, Moscow, Russia

**Keywords:** sudden cardiac death, monogenic canalopathy, long QT syndrome, Brugada syndrome, arrhythmic right ventricular dysplasia, implantable cardioverter-defibrillator

## Abstract

The abrupt cessation of effective cardiac function that is generally due to heart rhythm disorders can cause sudden and unexpected death at any age and is referred to as a syndrome called “sudden cardiac death” (SCD). Annually, about 400,000 cases of SCD occur in the United States alone. Less than 5% of the resuscitation techniques are effective. The prevalence of SCD in a population rises with age according to the prevalence of coronary artery disease, which is the most common cause of sudden cardiac arrest. However, there is a peak in SCD incidence for the age below 5 years, which is equal to 17 cases per 100,000 of the population. This peak is due to congenital monogenic arrhythmic canalopathies. Despite their relative rarity, these cases are obviously the most tragic. The immediate causes, or mechanisms, of SCD are comprehensive. Generally, it is arrhythmic death due to ventricular tachyarrythmias – sustained ventricular tachycardia (VT) or ventricular fibrillation (VF). Bradyarrhythmias and pulseless electrical activity account for no more than 40% of all registered cardiac arrests, and they are more often the outcome of the abovementioned arrhythmias. Our current understanding of the mechanisms responsible for SCD has emerged from decades of basic science investigation into the normal electrophysiology of the heart, the molecular physiology of cardiac ion channels, the fundamental cellular and tissue events associated with cardiac arrhythmias, and the molecular genetics of monogenic disorders of the heart rhythm (for example, the long QT syndrome). This review presents an overview of the molecular and genetic basis of SCD in the long QT syndrome, Brugada syndrome, short QT syndrome, catecholaminergic polymorphic ventricular tachycardia and idiopathic ventricular fibrillation, and arrhythmogenic right ventricular dysplasia, and sudden cardiac death prevention strategies by modern techniques (including implantable cardioverter-defibrillator)

## INTRODUCTION

The term “sudden cardiac death” (SCD) is used to denote death, presumably from cardiac causes, which occurs within 1 h after the onset of acute symptoms [1]. As a rule, the direct cause of such an outcome is cardiac arrhythmias: ventricular tachycardia (VT) and ventricular fibrillation (VF), which disrupt the pumping function of the heart leading to acute circulatory disorders and, in sufficient duration, to irreversible consequences with a fatal outcome. According to U.S. registers, the annual incidence of SCD in the United States is 50–00 per 100,000 of the population [2], or ca. 350–00,000 cases per year [3]. In Russia, 200–50,000 cases of SCD are registered each year [4].

The majority of SCD cases (75–80%) occur in adults and is associated with coronary artery disease (CAD). The period of acute myocardial infarction is the most susceptible to the development of ventricular arrhythmias. According to population studies, the incidence of SCD increases with age proportionally to the increase in CAD prevalence. For example, at the age below 35 years, the incidence of SCD is minimal (up to six cases per 100,000), and it gradually increases in middle and older age groups and reaches its maximum (346 cases per 100,000) for people aged 75 to 84 years. However, there is an additional peak in SCD incidence in children under the age of 5 (17 per 100,000), which is due to familial arrhythmogenic canalopathies [5].

The second most common cause of SCD is cardiomyopathies: hypertrophic cardiomyopathy and nonischemic dilated cardiomyopathy, which accounts for about 10–15% of all sudden arrhythmic deaths [6]. Infiltrative, inflammatory, and valvular heart diseases of different etiologies account for the majority of the remaining causes. Children and young adults are also susceptible to SCD usually due to genetic diseases, socalled canalopathies, which represent only a small portion of SCD causes (no more than 1–2%) [6].

Despite the different etiologies of sudden death, the causes behind this event are universal. As has already been mentioned, most commonly cardiac arrest is caused by sustained VT or VF. Primary pulseless electrical activity (PEA) or bradyarrhythmias is less common, accounting for no more than 40% of all SCD cases [5], and more often the two are outcomes of ventricular tachyarrhythmias. VT and/or VF hold the greatest potential for reversibility; only during this short period, until the transition to PEA or asystole, can normal heart rhythm be restored by electrical defibrillation. The need to capitalize on this “therapeutic window” dictates the need for the fastest possible diagnosis and immediate defibrillation.

The widespread use of implantable cardioverter-defibrillators (ICD) for both primary and secondary prevention of SCD has significantly reduced mortality in high-risk patients. Nevertheless, the incidence of SCD remains high. Even now, in an era of high-speed and new methods of information transfer, the survival rate after resuscitation does not exceed 5% in developed countries [6].

This review is dedicated to the rarest congenital causes of sudden death: monogenic arrhythmic disorders, as well as arrhythmogenic right ventricular dysplasia and modern approaches to sudden death risk stratification in these patients.

## CELLULAR BASIS OF ELECTROPHYSIOLOGY

The physiological processes of formation and propagation of electrical impulses in the heart muscle, as well as the “excitation-contraction” process, are remarkably fine-tuned and occur under the influence of harmonious workings of ion channels in accord with a variety of regulatory bioactive substances. Ion channels are proteins that enable selective permeability of the cell membrane for a particular ion. Voltage-gated ion channels open and close under the influence of the membrane potential, and ligand-gated ion channels require binding to an intra- or extracellular molecule to open an ion pore. In addition to ion channels, the intracellular homeostasis of ions is also maintained by ion pumps and exchangers that enable transmembrane transport of only certain ions with (pumps) or without (exchangers) use of ATP energy resources.

**Fig. 1:**
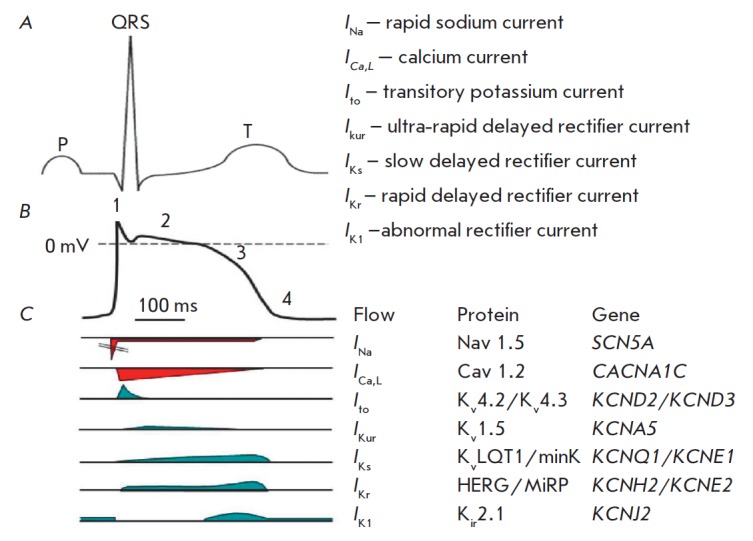
Approximate temporal relationships between surface ECG (A) and typical ventricular action potential (B) and ionic currents (C) through the membrane of cardiomyocyte. 0 – depolarization phase: 1 – rapid repolarization phase; 2 – plateau; 3 – repolarization; 4 – resting phase

The cardiomyocyte’s action potential is initiated by a regional change in the membrane potential that activates voltage-gated sodium (Na^+^) channels and initiate a fast but transient sodium current (*I*_Na_) that produces the typical ascending slope of the action potential curve known as Phase 0 depolarization (*Fig. 1*). Fast Phase 1 early repolarization is due to several ionic currents: the transient potassium current (K^+^), *I*to1 (transient outward), and the calcium-activated chloride current (Cl^-^),* I*_to_2 [7]. During Phases 0 and 1, Na^+^-channels are rapidly inactivated, whereas voltage-gated calcium (Ca^^2+^^) channels (L-type) are activated and participate in the formation of the sustained plateau of membrane depolarization. The plateau phase (Phase 2) is sustained by a delicate balance between the inward Ca^2+^ current (*I*_Ca_) through L-type channels, with the small residual Na current (*I*_Na_) and the emerging outflow K^+^ current. The activation of K^+^-channels, together with inactivation of Ca^2+^ channels, shifts this balance towards the outward currents, thereby initiating Phase 3 repolarization.

The outward potassium current (the so-called delayed rectifier current) consists of at least three components: ultra-rapid (*I*_Kur_), rapid (*I*_Kr_), and slow (*I*_Ks_), which differ in the rate of activation and pharmacological sensitivity [7]. These differences define the unequal duration of the action potential in different portions of the myocardium based on the level of channel expression [8]. The expression of genes encoding subunits of rapid K^+^-channels (*I*_Kr_ current), *KCNH2*, is subject to pronounced diurnal variation, playing the role of a “molecular clock” of sort. Disruptions of the circadian clock mechanism may be associated with an increased risk of sudden death [9].

Finally, an abnormal inward rectifier current (*I*_K_1) completes the process of cardiomyocyte membrane repolarization. This current is called abnormal because its formative K^+^-channels are activated only in the case of negative charge of the membrane potential and enable, primarily, an inward current.

**Fig. 2:**
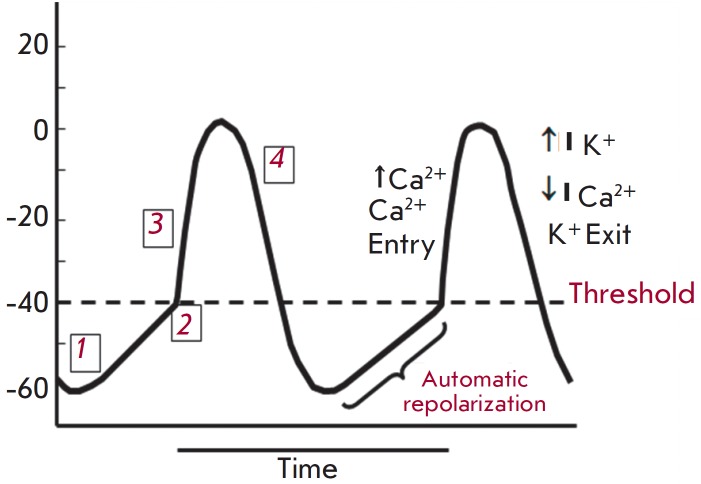
Action potential of pacemaker cell. 1 – automatic depolarization – I_f_ channels are open; 2 – membrane potential reaches the threshold level – transient T-type calcium channels are opening; 3 – slow (L-type) calcium channels are opening – depolarization; 4 – L-type calcium channels are closing, potassium channels are opening – hyperpolarization

Pacemaker myocardial cells (*Fig. 2*) possess a special mechanism for the action potential buildup, which can spontaneously generate the action potential. Even the cardiomyocytes of the sino-atrial node that are isolated from all surrounding tissues maintain spontaneous diastolic depolarization. [7]. This ability is enabled through a special ion flow, called “funny” –*I*_f_ –due to its unusual properties. The *I*_f_ current is a mixed inward calcium-sodium current which is gradually initiated during the hyperpolarization (after the completion of Phase 4 repolarization) at a transmembrane potential of –40/–50 mV and is fully activated at a potential of about –100 mV, initiating the action potential. The pacemaker current is implemented through a family of ion channels discovered in the 1990s and called HCN channels (hyperpolarization-activated channels). The autonomic modulation of the pacemaker current has undeniable significance for normal physiology of cardiac activity and is implemented through cAMP. There are four isoforms of HCN-channels which differ in the rate of activation and inactivation, as well as in sensitivity to cAMP. Experiments have shown that adrenergic and cholinergic neurotransmitters cause an increase or decrease in the level of intracellular cAMP, respectively. Subsequently, cAMP binds directly to the HCN-channel, strengthening or weakening the *I*f current, resulting in acceleration or deceleration of spontaneous depolarization.

Ion exchangers and pumps play a crucial role in the disposal of the excess of ions arising during the formation of each action potential, as well as in the maintenance of exact levels of ions within the cell. The two most studied ion pumps are the membranes Na^+^-K^+^-ATPase and Ca^2+^-ATP-ase and the two most studied ion exchangers are the membranes Na^+^-Ca^2+^ and Na^+^-H^+^.

Na^+^-K^+^-ATP-ase is a magnesium-activated (Mg^2+^) cardiomyocyte membrane enzyme. Under physiological conditions, the pump maintains a normal resting potential, ensuring the transfer of three Na^+^ ions out of the cell in exchange for the influx of two K^+^ ions into the cell. The function of the Na^+^-pump is critical in maintaining the level of intracellular Na^+^, and, consequently, it affects cardiomyocyte contractility and excitability [7].

The membrane Ca^2+^ pump (ATPase), together with the Na^+^-Ca^2+^ exchanger, removes intracellular Ca^2+^. However, its role in the utilization of Ca^2+^ is presumably small. Ca^2+^-ATP-ase of the sarcoplasmic reticulum is much more important. The primary role in removing excess intracellular Ca^2+^ belongs to the Na^+^-Ca^2+^ exchanger. Switching-off of the gene encoding the sarcolemmal protein leads to the death of an embryo on Days 9–10 after conception [7]. The Na^+^-Ca^2+^ exchanger is a quantitative transfer protein providing transport of Na+ in exchange for Ca^2+^ (3:1). However, the function of the Na^+^-Ca^2+^ exchanger is coordinated with the ever- changing inward flow of Ca^2+^: the system removes the exact amount of Ca^2+^ that has entered into the cell during the ongoing cardiac cycle. Two systems – the Na^+^-Ca^2+^ exchanger and the sarcoplasmic reticulum (SPR) – participate in the removal of Ca^2+^ from the cytoplasm during the relaxation of the myocardium. Experiments have proven that each system alone is able to fully enable myocardial relaxation. Ca^2+^-ATP-ase of the sarcoplasmic reticulum can ensure rapid relaxation of the heart muscle, but it is unable to work on its own over several consecutive contractions. In contrast, the Na^+^-Ca^2+^ exchanger enables repeated outflow of Ca^2+^ from one contraction to another. Depending on the electrochemical gradients, the Na+-Ca^2+^ exchanger is able to provide not only the outflow of Ca^2+^, but also the inward Ca^2+^ current in the cell to maintain or enhance myocardial contractility.

The Na^+^-H^+^-exchanger is also a quantitative transfer protein; it replaces one intracellular proton with one extracellular sodium ion and plays an essential role in the maintenance of intracellular pH.

Ion channels function is regulated by a variety of intra- and extracardiac factors, the most significant of which is β-adrenergic stimulation. For example, upon physiological activation of the sympathetic nervous system due to physical exertion or emotional stress, known in English literature as “fight or flight” reaction, the increased heart rate requires immediate shortening of the cardiomyocytes action potential, which is implemented through an increase of *I*_Ks_ via β-adrenergic stimulation [7]. In addition, the sympathetic stimulation enhances myocardial contractility, mainly through an increase in the inward Ca^2+^ current and increased accumulation of Ca^2+^ in the sarcoplasmic reticulum, for subsequent enhanced release inside the cell.

Intracellular Ca^2+^ homeostasis mainly depends on normal operation of the SPR. Voltage-gated Ca^2+^-channels in the SPR membrane are regulated by the socalled ryanodine receptors, RyR2, whose dysfunction can lead to cell overload with Ca^2+^ and subsequent increase in triggering of myocardial activity.

## MONOGENIC CAUSES OF SUDDEN CARDIAC DEATH

The symphony of ion channel performance is disturbed by genetically predetermined ion canalopathies. Such defects can trigger fatal arrhythmias, which usually occur in childhood or at a young age. Despite a low incidence, these diseases can be identified by molecular diagnostics, which have allowed to elucidate the most frequent causes of these genetic abnormalities over almost two decades. Currently, more than 25 genes are known whose disruption of expression can cause susceptibility to ventricular tachyarrhythmias. Only few isolated nosological forms are identified clinically. The main ones are long QT syndrome (LQTS), short QT syndrome (SQTS), Brugada syndrome (BS), catecholaminergic polymorphic ventricular tachycardia (CPVT), and idiopathic VF. These diseases are based on three pathophysiological mechanisms: impaired repolarization (LQTS, SQTS, Brugada syndrome), delayed ventricular conduction (Brugada syndrome), and disruption of intracellular Ca^2+^ homeostasis (CPVT).

**Congenital long QT syndrome (LQTS)**

The most common variant of LQTS occurs in the Romano- Ward syndrome, which is inherited through an autosomal dominant mechanism (incidence of about 1 in 2,500 live births) [10]. A less frequent variant is the Jervell-Lange-Nielsen syndrome, which is autosomal- recessive and combined with deafness. Genetically, LQTS is very heterogeneous; there are at least 8 identified variants. The most common genetic subtype is LQTS1, which is caused by mutations in the *KCNQ1* gene that encodes a subunit of the voltage-gated K^+^ channel responsible for the slow outward K^+^ current (*I*_Ks_). Mutations in the *KCNH2 *gene that defines the structure of another version of the K^+^ channel subunit responsible for a rapid outward K^+^ current (*I*Kr) result in the development of the second major subtype, LQTS2. Heterozygous mutations in *KCNQ1 *and* KCNH2 *cause a loss of function by the respective channels, decreasing *I*_Ks_ or *I*_Kr_, respectively, which slows down the repolarization and prolong the ventricular action potential. An increased heart rate during sympathetic activation reveals the inability of the cardiomyocytes in such people to increase *I*_Ks_. It explains the fact that exercise and emotional stress provoke the onset of life-threatening arrhythmias in patients with LQTS1. At the same time, trigger factors for patients with LQTS2 are sharp acoustic stimuli (cry, alarm clock, etc.) [11]. The genetic specificity of arrhythmogenic triggers has been demonstrated in a sample of 700 patients with a known LQTS genotype. For example, 99% of arrhythmic events during swimming occurred in patients with LQTS1, whereas 80% of the events provoked by sudden sounds occurred in patients with LQTS2 [12].

Ca. 10% of all LQTS are caused by mutations in the* SCN5A *gene (LQTS3), which encodes the α-subunit of the Na^+^-channel, which enables a rapid inward Na^+^ current during Phase 0 depolarization. Typically, these are gains of function mutations that disrupt channel inactivation and increase in a constant *I*_Na_ [13]. A similar phenotype has been observed for the mutations in other genes (including *CAV3*, *SCN4B, *and *SNTA1*) which encode proteins that directly or indirectly affect sodium channels. A constantly elevated Na^+^ current disrupts the physiological balance between inward and outward ions flows during the plateau phase, causing delayed repolarization, prolongation of the action potential, and predisposition to re-entry arrhythmias. Selective blockade of the constant Na^+^ current by some antiarrhythmic drugs (such as mexiletine) or the antianginal drug ranolazine may serve as a pathophysiologically based approach to LQTS3 treatment [14, 15]. It should be noted that the *KCNQ1*, *KCNH2, *and *SCN5A* genes, mutations in which cause LQTS 1, 2 and 3, respectively, are so-called “major” LQTS genes, and mutations in them imply a high probability of congenital LQTS and is important for risk stratification (see below).

Acquired LQTS are more common than congenital ones, and they have very similar pathophysiological mechanisms. The most common variant of acquired LQTS is medical prolongation of the QT interval that occurs when cardiac or noncardiac medications are used to block the K^+^ channels that enable* I*_Kr_ (HERG-channel), which leads to a slowing-down of ventricular repolarization. There are also variants of genetic predisposition to drug-related prolongation of the QT interval [16]. These conditions are associated with partial loss of function in respect to *I*_Ks_, which leads to a decrease in the so-called repolarization reserve that can manifest itself in the case of *I*_Kr_ inhibition with drugs. There are also individual cases of manifestation of latent congenital LQTS in patients receiving drugs that block HERG-channels (e.g., antiarrhythmic drugs such as amiodarone, sotalol, dofetilide, propafenone) [13], or in case of other pathological conditions, such as myocardial infarction [17].

In addition to the Jervell-Lange-Nielsen syndrome, there are two other types of LQTS with extracardiac manifestations. The Andersen syndrome is an autosomal dominant disease characterized by ventricular arrhythmias, periodic paralysis, and bone manifestations [12]. Anderson syndrome is phenotypically heterogeneous, often with one or two clinical signs. Although ventricular arrhythmias can be classified as major manifestations of the disease, they rarely result in sudden death [13]. Andersen syndrome is associated with a mutation in the *KCNJ2 *gene that encodes the K^+^-channel which enables an abnormal inward rectifier current,* I*_K1_, an important component of Phase 3 repolarization. Disruption of the channel function leads to a lengthening of the action potential and increased tendency towards re-entry.

The Timothy syndrome is associated with a mutation in the *CACNA1C *gene that encodes the subunit of the voltage-gated Ca^2+^ channel. Symptoms of Timothy syndrome include heart rhythm abnormalities, syndactyly, and autism [13]. The mutation causes pronounced disruption of Ca^2+^ channel inactivation and an excessive Ca^2+^ current during the plateau phase.

**Fig. 3:**
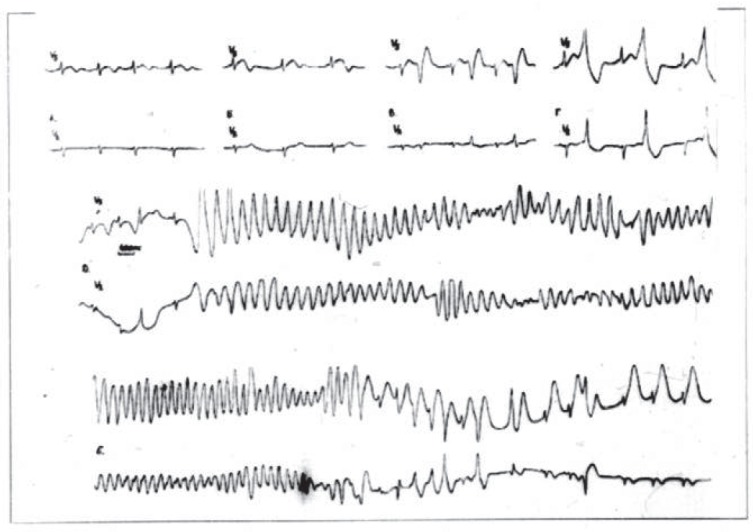
Fragment of ECG Holter monitoring of a patient, female, 13 y.o., diagnosis congenital long QT syndrome. Paroxysm of torsades de pointes (own data).

LQTS is characterized by a particular electrocardiographic pattern immediately prior to the ventricular tachycardia, the so-called short-long-short sequence (SLS) or “cascade” phenomenon, which includes alternation of shortening of RR intervals due to supraventricular premature contraction (short), followed by post-premature contraction pause (long) and repeated ventricular premature contraction (short), with subsequent “torsades de pointes” tachycardia (*Fig. 3*) [12, 18]. In 2011, P. Schwartz presented updated diagnostic criteria for LQTS (*Table 1*). A total score of ≥3.5 justifies a LQTS diagnosis (in the absence of secondary causes) [19-21]. Furthermore, a LQTS diagnosis can be established by identifying tcharacteristic genetic mutation, regardless of the duration of the QT interval [21].

**Table 1:** Diagnostic criteria for long QT syndrome. P. Schwartz score (2011) [19]

Criteria	Points
QTc > 480 ms	3
QTc = 460–470 msc	2
QTc = 450 ms (men)	1
QTc 4th minute of recovery from exercise stress test ≥ 480 ms	1
Torsades-de-Pointes	2
T-wave alternans	1
Notched T wave in 3 leads	1
Low heart rate for age	0.5
Stress-induced syncope	2
Stress-free syncope	1
Congenital deafness	0.5
Family members with definite LQTS	1
Unexplained sudden cardiac death younger than age 30 among immediate family members	0.5

**Fig. 4:**
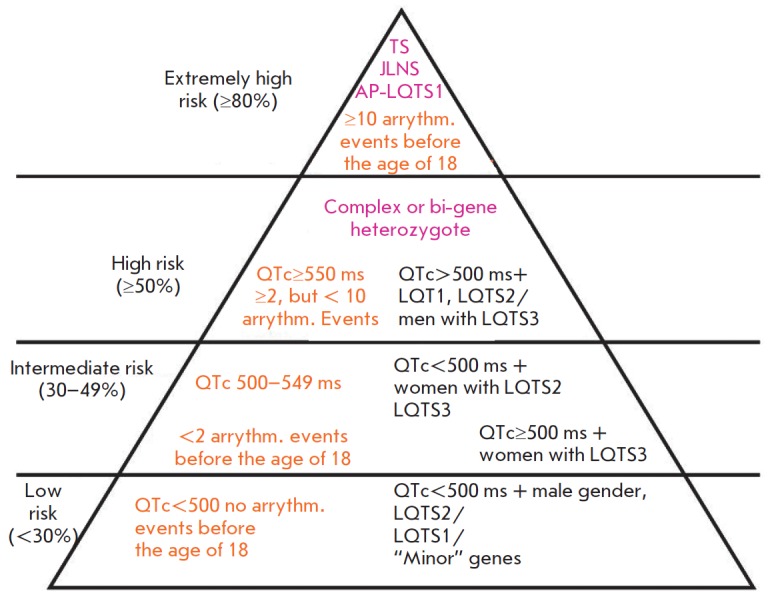
Genotype- and phenotype- guided risk classification of long QT syndrome patients. Phenotype-guided recommendations are indicated by orange text, Genotype-guided recommendations are indicated by purple text, and a combination of genotype- and phenotype- guided recommendations is indicated by black text within the figure. LQTS, long QT syndrome; Arrythm. events, arrhythmic events; AP-LQTS1, autosomal recessive LQTS; JLNS, Jervell- Lange-Nielsen syndrome; TS, Timothy syndrome.

Both the genetic status and clinical data are important for a stratification of the risk of arrhythmic events in patients with LQTS. Researchers at the Mayo Clinic Giudicessi J.R. *et al*. [22] have developed a risk stratification scheme for primary and recurrent cardiac events, including syncope, sudden cardiac arrest or sudden cardiac death before the age of 40 years, based on recent studies that examined adverse events in LQTS patients (*Fig. 4*).

Currently, beta-blockers are the only class of drugs recommended for patients with LQTS - [22, 23]. They are particularly effective in LQTS1 patients whose trigger factors are physical exercise and whose tone of the sympathetic nervous system is significantly elevated. The protective effect of beta-blockers is less pronounced in LQTS2 and LQTS3.

In addition to drug therapy, the frequency of arrhythmic events in LQTS patients can be reduced by extrapleural or thoracoscopic left-sided cardiac sympathectomy, including removal of the lower half of the stellate ganglion (T1) and thoracic ganglia (T2–T4). Schwartz *et al*. have demonstrated a decrease in the frequency of arrhythmic events by more than 90% within 8 years after surgical denervation in a group of 147 high-risk LQTS patients (average QTc 563 Ѓ} 65 ms; 99% symptomatic) [24].Modern concepts suggest the use of left-sided cardiac sympathectomy in patients who cannot tolerate β-blockers or for whom they are inefficient [22].

The decision about implantation of a cardioverter- defibrillator (ICD) should be made on an individual basis. According to the observation of 233 LQTS patients within < 5 years after ICD implantation, 28% of them underwent efficient electrotherapy. At the same time, at least 31% of them had at least one post-implantation complication [25]. In 2012, Schwartz *et al*. developed a clinical M-FACT scale to identify patients in need of ICD (*Table 2*). According to the authors, implantation of ICD is justified at a score of ≥ 1 point.

**Table 2:** M-FACT risk scale for a decision on implantation of cardioverter-defibrillator in patients with long QT syndrome (Schwartz et al., 2012) [26]

Criteria	– 1 point	0 points	1 point	2 points
Event free on therapy for >10 y	Yes			
QTc, ms		≤ 500	> 500 ≤ 550	> 550
Prior ACA		No	Yes	
Events on therapy		No	Yes	
Age at implant, years		> 20	≤ 20	

Note. M-FACT is deciphered as M for Minus 1 point for being free of cardiac events, while on therapy for >10 y; F for Five hundred and Five hundred and Fifty millisecond QTc; A for Age ≤20 y at implant; C for Cardiac arrest; T for events on Therapy; ACA, aborted cardiac arrest.

**Short QT interval syndrome (SQTS)**

The short QT interval syndrome (SQTS) was first described as late as in 2000. This repolarization disorder is characterized by QT shortened to 320 ms and lower, high T-wave, and relative increase in the interval between the peak and the end of the T-wave [27]. However, according to population studies, shortening of the QT interval does not always imply true congenital SQTS and is not always accompanied by susceptibility to life-threatening arrhythmias [28]. In addition to a consistently shortened QT, patients with congenital SQTS are characterized by shortening of the ST segment up to its complete absence and start of the T-wave directly from the S-wave.

The shortening of the QT interval, as well as its prolongation, is associated with life-threatening arrhythmias and SCD, often in childhood. Mutations in six different genes encoding subunits of the K^+^ (*KCNQ1*,* KCNH2*, *KCNJ2*) or Ca^2+^ (*CACNA1C*, *CACNB2*, *CACNA2D1*) channel have been identified as associated with this phenotype. Many of these genes are similar to those implicated in LQTS: however, the functional outcome of the mutations is exactly the opposite. Gainof- function mutations of K^+^-channels genes lead to increased repolarization and shortening of the action potential. Mutations in Ca^2+^-channels genes, on the contrary, lead to loss-of-function.

A diagnosis of SQTS can be established at QTc ≤ 340 ms. At QTc ≤ 360 ms, the diagnosis is valid in the presence of characteristic genetic mutations, family history of SQTS, familial cases of sudden death at an age < 40 years or VT/VF episodes without cardiac pathology [21].

According to the latest European guidelines, implantation of a cardioverter-defibrillator is advised only as a secondary prevention. Sotalol or quinidine can be used as antiarrhythmic therapy (recommendation grade IIb) [21].

**Brugada syndrome (BS)**

**Fig. 5:**
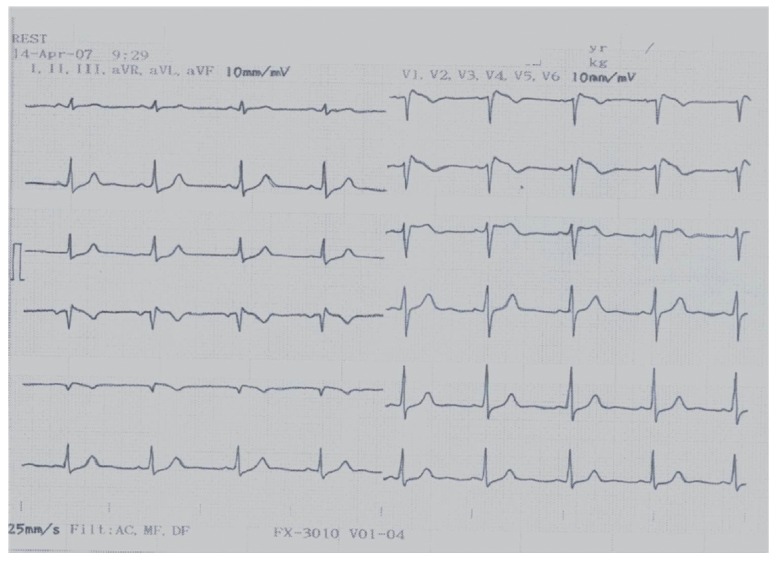
ECG of a patient with Brugada syndrome (own data)

Patients with the Brugada syndrome are prone to developing fatal arrhythmias mainly during sleep, in the absence of myocardial ischemia, electrolyte abnormalities, and structural heart diseases [13]. Changes in resting ECG characteristics for BS patients are wellknown: ST-segment elevation in the right precordial leads, signs of a right bundle branch blockage combined with normal duration of the QT interval (*Fig. 5*). Prescription of Na^+^-channels blockers (procainamide, flecainide, ajmaline), as well as fever, may reveal hidden ECG disorders. Cases of unexplained sudden death in the family history are quite typical. The prevalence of the Brugada syndrome in Europe and America is about 1:10,000 of population [13].

A total of 350 different variants of gene mutations associated with BS [29] [30] has been described to date: in 30% of cases, they are mutations in the *SCN5A *gene that encodes the α-subunit of rapid Na+-channels; in 5%, mutations in other genes, including those encoding Ca^2+^- and K^+^ channels proteins; and in 65% of cases, the genetic substrate is not identified [31, 32]. *SCN5A* gene mutations lead to a decrease in the number of Na+-channels and acceleration of their inactivation in the right ventricular epicardium cells, which locally reduces* I*_Na_ in the epicardium. The resulting disruption of ventricle wall repolarization leads to a transmural voltage gradient, which manifests itself at ECG as ST-segment elevation and serves as a substrate for re-entry into the ventricular myocardium [33].

According to other researchers, the inhibition of the inward sodium current slows down pulse conduction in the right ventricle, causing delayed activation of the myocardium in the right ventricular exit sites. This leads to asynchronous depolarization and electrical instability in this section of the heart with possible development of ventricular arrhythmias by the re-entry mechanism [13, 34]. It is unclear whether these two hypotheses are mutually exclusive or if all BS variants are subject to a single pathophysiological mechanism.

A genetic analysis to identify mutations in genes typical for BS is useful for verification of the diagnosis, but it has no independent value in the risk stratification. Moreover, the absence of mutations does not preclude the diagnosis

Risk stratification in asymptomatic patients with Brugada syndrome is the most important and, at the same time, controversial issue. The annual incidence of cardiac arrest or syncopes in BS patients with a history of sustained VT or VF is between 1.9 [35] and 8.8 [36], 7.7 [37] and 13.8% [38], respectively. Implantation of ICD to such patients is the only truly effective treatment [37]. However, most patients (64% according to a large-scale FINGER study) [35] have no clinical manifestations of the disease at the time of verification of the diagnosis. The incidence of arrhythmic events in such patients is significantly lower and ranges from 0 to 0.8% (0.5% according to FINGER data) [37]. On the other hand, the young age of these patients and the absence of structural heart diseases suggest that a low annual risk of cardiac events is only temporary and will increase in the subsequent few decades.

The role of programmed ventricular stimulation in invasive EPS for risk stratification in asymptomatic patients has been actively discussed since BS was described for the first time. Recent studies, including the largest ones, FINGER and PRELUDE, found no independent effect of invasive electrophysiologic studies on arrhythmic events over an average of 32 and 18 months [35, 38]. However, according to a recently published meta-analysis of 14 studies, which included 3,536 patients with an asymptomatic Brugada syndrome phenotype, a typical spontaneous pattern of type 1 ECG (i.e. ST elevation in the right precordial leads of more than 2 mm with a negative T-wave and J-wave) (*Fig. 5*), as well as the induction of ventricular tachyarrhythmias by the programmed ventricular stimulation, increases the risk of future arrhythmic events. The length of the follow-up period was 20 to 77 months [39]. Therefore, at the moment the recommendation of ICD implantation on the basis of EPS data has class of recommendation IIb; i.e., “can be considered” for the induction of VF during the programmed ventricular stimulation with two or three extrastimules in two points (Clinical Recommendations for Diagnosis and Treatment of Ventricular Arrhythmias, European Society of Cardiology, 2015) [21].

In other cases, ICD implantation is indicated for BS patients as secondary prevention (class of recommendation I), and it should be considered in case of a spontaneous manifestation of type I ECG and history of syncope of unknown origin (class of recommendation IIa) [21]. Quinidine and isoproterenol are recommended as preventive antiarrhythmic therapy, including for the treatment of “electrical storm” (class of recommendation IIa). In addition, BS patients are advised to observe a number of rules to minimize the known factors that trigger arrhythmia, such as excluding administration of drugs that can aggravate ST elevation in the right precordial leads, avoiding excessive use of alcohol and heavy meals, and using antipyretics in a fever of any origin as soon as possible [21].

**Catecholaminergic polymorphic ventricular tachycardia (CPVT)**

Disruption of intracellular Ca^2+^ homeostasis leads to serious arrhythmogenic effects. Mutations in the *RyR2* gene encoding the ryanodine receptors that are responsible for the release of calcium from the sarcoplasmic reticulum of the cardiomyocyte cause the development of an autosomal dominant variant of catecholaminergic polymorphic ventricular tachycardia. Autosomal recessive types of the disorder are caused by impairments of the *CASQ2 *gene function, which encodes the calsequestrin protein that binds Ca^2+^ of the sarcoplasmic reticulum, or mutations in the *TRDN *gene that encodes triadin which binds calsequestrin to RyR2-receptors [13]. These three proteins are located in the terminal SPR cistern, where the intracellular membrane is in close proximity to the region of transverse tubulae (T-tubulae) of the plasma membrane. Normally, electrical pulses are delivered into the T-tubulae system and activate L-type Ca^2+^-channels, causing fluctuations in Ca^2+^ concentration sufficient to cause a Ca^2+^-induced release of Ca^2+^ via ryanodine receptors. Release of Ca^2+^ from the SPR causes contraction of the myocyte, which ends in the removal of Ca^2+^ from the cytosol, mainly via the Ca^2+^-ATP-ase and Na^+^/Ca^2+^ pumps. Disruption of the function of these receptors leads to cardiomyocyte overload with Ca^2+^, electrical instability of the cells, and the formation of post-depolarization potentials. Catecholamines that enter the blood in time of stress and/ or exercise cause contraction of the heart muscle via phosphorylation of protein kinase of the ryanodine receptor [40-42].

Other genes associated with polymorphic VT have also been identified. It is believed that mutation in the* KNJ2 *gene, which is associated with the development of the Andersen syndrome, may be the cause of familial catecholaminergic VT. There are reports on mutations in the ankyrin B gene, which are also present in LQTS4. Recently, it has been suggested that idiopathic ventricular fibrillation can be a form of familial polymorphic ventricular tachycardia [43].

The diagnosis of catecholaminergic polymorphic VT is basically an exclusion diagnosis in which bi-directional VT or VF in response to physical or emotional stress occurs in patients without a structural heart disease and changes in resting ECG.

Drug therapy consists of the prescription of beta- blockers. There are several reports on the effectiveness of calcium channel blockers (verapamil) in familial polymorphic ventricular tachycardia. In general, a lifestyle change is indicated to all patients, including exclusion of physical activities and exercise.

**Idiopathic ventricular fibrillation**

Idiopathic ventricular fibrillation is a rare disease of unknown etiology, which manifests itself as syncope and SCD in the absence of data in favor of an organic heart disease or canalopathy. Idiopathic VF is characterized by spontaneous development of fatal arrhythmia unrelated to physical stress, often during sleep. VF is initiated by premature ventricular contraction with a very short coupling interval. It has been demonstrated that Purkinje fibers are involved in the induction and maintenance of arrhythmia with a re-entry mechanism [44].

**Arrhythmogenic right ventricular dysplasia (ARVD)**

Arrhythmogenic right ventricular dysplasia (or cardiomyopathy) (ARVD) is a rare inherited disease characterized by ventricular arrhythmias, sudden cardiac death, and dysfunction of the right ventricle. More than 30 years have passed since the first detailed description of ARVD in 1982. Numerous clinical and experimental studies of this disease have been published since. For example, it has been established that the most common genetic causes of ARVD are mutations in desmosome proteins, the basic elements of cell-cell adhesion structures present in the multilayered epithelium and the myocardium; e.g., the results of a recently published study involving 577 patients in the U.S. (Johns Hopkins registry) and Danish ARVD registers showed that 80% of patients had a mutation in the *PKP2 *gene encoding plakophilin, one of the desmosomal proteins. The remaining participants in the study had mutations in other desmosomal protein genes: *DSG2 *(desmoglein), *PLN* (plakophilin), *DSP *(desmoplakin), *DSC2 *(desmocollin),* JUP *(junction plakoglobin), *TMEM43 *(transmembrane protein 43) [45].

This myocardium dysplasia can be called “cardiomyopathy of intercellular contacts” [11]. Defective desmosomal proteins disrupt the mechanical connection between adjacent muscle cells, which leads to their separation, especially in the context of myocardial stretching. The accompanying inflammation, fibrosis, and adipocytosis may be a nonspecific response to damage, similar to the one caused by any damage to the myocardium [46]. This pathogenetic model explains the fact that prolonged excessive stress, accompanied by myocardial stretching, significantly increases the risk of early clinical manifestation of the disease and increases the risk of SCD. In addition, it explains why the pathological ARVD process often involves the more stretchable and thin-walled right ventricle, especially in the early stages of the disease. Naturally, the mechanical separation leads to electrical heterogeneity, forming an ideal substrate for the development of ventricular re-entry tachycardia [11].

**Fig. 6:**
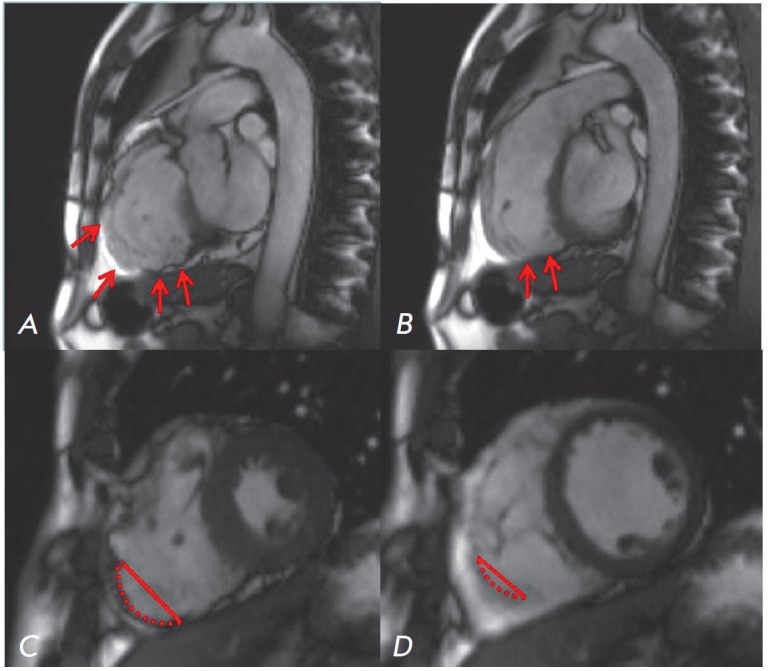
Cardiac MRI of a patient with ARVD (own data). Bright blood images in the right ventricular outflow tract (RVOT) plane obtained in end diastole (A) and end systole (B) show microaneurysms (arrows) in the right ventricle free wall with persistent bulge in both phases. Short-axis bright blood images obtained in end diastole (C) and end systole (D) demonstrate dyskinesis (bulge in systole, arrows) at the acute angle of the right ventricle

Task Force Criteria (TFC) for the diagnosis of ARVD were proposed in 1994 and revised in 2010 [47]. The diagnostic criteria for ARVD include characteristic changes in depolarization/repolarization on the electrocardiogram, echocardiography, and magnetic resonance imaging (MRI) data (*Fig. 6*) describing changes in morphology and function of the right ventricle, characteristic changes in myocardial tissue observed in endomyocardial biopsy, as well as the presence of ventricular arrhythmias, details of family history and genetic testing (*Table 3*). To justify an ARVD diagnosis, a patient must score four points, with one major criterion being worth two points, and one minor criterion being worth one point. ARVD is considered to be “probable” at three points, whereas a score between one and two should be considered as an absence of ARVD [47].

**Table 3:** Diagnostic criteria for arrhythmogenic right ventricular dysplasia (ARVD) (F. Marcus et al., 2010) [47]

Group	Major criterion	Minor criterion
Global or regional dysfunction and structural alterations	**By 2D echo:** Regional RV akinesia, dyskinesia, or aneurysm AND 1 of the following (end diastole): PLAX RVOT 32 mm PSAX RVOT 36 mm OR fractional area change 33%; **By MRI:** Regional RV akinesia or dyskinesia or dyssynchronous RV contraction AND 1 of the following: Ratio of RV end-diastolic volume to BSA 110 mL/m2 (male) or 100 mL/m2 (female) RVEF ≤ 40% **By RV angiography:** Regional RV akinesia, dyskinesia, or aneurysm	**By 2D echo:** Regional RV akinesia or dyskinesia or dyssynchronous RV contraction AND 1 of the following (end diastole): PLAX RVOT 29 to 32 mm; PSAX RVOT 32 to 36 mm OR fractional area change 33% to 40%; **By MRI:** Regional RV akinesia or dyskinesia or dyssynchronous RV contraction AND 1 of the following: Ratio of RV end-diastolic volume to BSA 100 to 110 mL/m2 (male) or 90 to 100 mL/m2 (female); RVEF> 40 ≤45%
Tissue characterization of wall	Residual myocytes 60% by morphometric analysis (or 50% if estimated), with fibrous replacement of the RV free wall myocardium in 1 sample, with or without fatty replacement of tissue on endomyocardial biopsy	Residual myocytes 60% to 75% by morphometric analysis (or 50% to 65% if estimated), with fibrous replacement of the RV free wall myocardium in 1 sample, with or without fatty replacement of tissue on endomyocardial biopsy
Repolarization abnormalities	Inverted T waves in right precordial leads (V_1_, V_2_, and V_3_) or beyond in individuals 14 years of age (in the absence of complete right bundle-branch block)	Inverted T waves in leads V_1_ and V_2_ in individuals 14 years of age (in the absence of complete right bundle-branch block) or in V_4_, V_5_, or V_6_ Inverted T waves in leads V_1_, V_2_, V_3_, and V_4_ in individuals 14 years of age in the presence of complete right bundle-branch block
Depolarization/ conduction abnormalities	Epsilon wave (reproducible low-amplitude signals between end of QRS complex to onset of the T wave) in the right precordial leads (V_1_ to V_3_)	Late potentials by SAECG in 1 of 3 parameters in the absence of a QRS duration of 110 ms on the standard ECG: fQRS ≥ 114 ms Duration of terminal QRS 40 µV (low-amplitude signal duration) 38 ms Root-mean-square voltage of terminal 40 ms QRS ≤ 20 ms Terminal activation duration of QRS 55 ms measured from the nadir of the S wave to the end of the QRS, including R’, in V_1_, V_2_, or V_3_, in the absence of complete right bundle-branch block
Arrhythmias	Nonsustained or sustained ventricular tachycardia of left bundle-branch morphology with superior axis (negative or indeterminate QRS in leads II, III, and aVF and positive in lead aVL)	Nonsustained or sustained ventricular tachycardia of RV outflow configuration, left bundle-branch block morphology with inferior axis (positive QRS in leads II, III, and aVF and negative in lead aVL) or of unknown axis 500 ventricular extrasystoles per 24 hours (Holter)
Family history	ARVC/D confirmed in a first-degree relative who meets current Task Force criteria ARVC/D confirmed pathologically at autopsy or surgery in a first-degree relative Identification of a pathogenic mutation† categorized as associated or probably associated with ARVC/D in the patient under evaluation	History of ARVC/D in a first-degree relative in whom it is not possible or practical to determine whether the family member meets current Task Force criteria Premature sudden death (35 years of age) due to suspected ARVC/D in a first-degree relative ARVC/D confirmed pathologically or by current Task Force Criteria in second-degree relative

BSA, body surface area; RVOT, RV outflow tract; PVC, premature ventricular contraction; EDV RV, end-diastolic volume of the right ventricle; LBBB - left bundle branch block; RV – right ventricle; PLAX parasternal long-axis view; PSAX, parasternal short-axis view; RVEF, right ventricle ejection fraction; CM – Holter ECG monitoring; Echo, echocardiography; EMB – endomyocardial biopsy.

Proper treatment of ARVD patients largely depends on an adequate diagnosis. Treatment is defined by the following strategies: SCD risk stratification and addressing the issue of implanting a cardioverter-defibrillator (ICD), minimizing the frequency of ICD discharges, and prevention of the progression of the disease. According to the general recommendations for SCD prevention, ICD implantation in ARVD is indicated in patients who have had ventricular fibrillation, or sustained ventricular tachycardia or syncope. The study by Bhonsale A. *et al*. included 84 patients with ARVD who were followed for 4.7 ± 3.4 years after ICD implantation as primary prevention. The predictors of effective electrotherapy were the symptoms (i.e. the status of the subject and not that of a family member), induction of VT at electrophysiological study, presence of unstable ventricular tachycardia, and more than 1,000 premature ventricular contractions (PVC) per day. The induction of ventricular tachyarrhythmias at EPS was an independent risk factor for the effective discharge of ICD [48].

Therefore, currently ICD implantation is indicated for patients who meet TFC criteria, especially if they have a history of SCD, sustained VT or arrhythmogenic syncope, a high number of PVCs, and/or unstable VT [46]. Clinicians should be especially attentive to SCD risk stratification in patients for whom ARVD was identified during a family screening. Typically, such patients are at earlier stages of the disease. Limitation on physical activity and the use of β-blockers can reduce the risk of SCD in such individuals. However, careful monitoring of the patient’s condition is recommended for all patients with a decision not to implant ICD.

β-blockers are indicated for all patients with ARVD. Amiodarone, or sotalol is recommended as additional antiarrhythmic therapy. In rare cases, other antiarrhythmic agents are used. If antiarrhythmics and repetitive ICD discharges are ineffective, it is recommended to perform radiofrequency ablation of arrhythmogenic foci. It should be noted that the role of catheter ablation in patients with ARVD is limited to a possible reduction in the number of defibrillator discharges and improved quality of life. According to several studies on the effectiveness of catheter ablation in patients with ARVD, only 25 to 47% were VT-free during the first year of observation, and 5 and 10 years after surgery the numbers were 21 and 15% [46]. Efficacy of epicardial ablation is slightly higher and amounts to 64% during the first year and 45% after 5 years [46]. According to Philips B. *et al*., in 30 patients with ARVD there were no effective ICD discharges after epicardial radiofrequency ablation in 83, 76, and 70% of cases for 6, 12, and 24 months, respectively [49].

The only currently available effective way to slow the progression of the disease is limitation on physical activity.

## CONCLUSION

In conclusion, we would like to present the survey data published in 2014 by the European Heart Rhythm Association, which included cardiologists from 50 clinics in 23 countries [50]. The survey focused on the diagnosis and treatment of patients with congenital arrhythmic syndromes. According to the study, most patients with canalopathies undergo genetic testing: from 70% among LQTS to 36% among patients with idiopathic VF. Although only a third of clinicians discuss the test results with patients and specialists in genetics, pharmacological tests are relatively frequently used for the diagnosis of congenital canalopathies. For example, 89% of the respondents used sodium channel blockers for the diagnosis of the Brugada syndrome, and 36% used isoproterenol to confirm catecholaminergic ventricular tachycardia. 80–92% of the doctors do not use pharmacological provocation for the diagnosis of the remaining canalopathies. Most clinics (82–98%) do not resort to intracardiac EPS to induce ventricular arrhythmias, with the exception of BS cases (39% of clinics use EFI). From 27 to 54% of the study participants included MRI in the diagnostic protocol for patients with BS and idiopathic ventricular arrhythmias, but only rarely for patients with LQTS and SQTS (11–17% of participants). Coronary angiography is performed in 62% of cases of idiopathic VF/VT. Endomyocardial biopsy is included in the study protocol of 8% of the patients with idiopathic VF. In most clinical centers, ICD implantation for primary prevention is performed only in 0–5% of patients with congenital canalopathies, whereas ICD usage for secondary prevention increases to 90–100%. Recurrent ventricular arrhythmias, leading to multiple ICD discharges, are treated with intensification of therapy, the use of β-blockers, and various antiarrhythmic drugs (isoproterenol infusion, quinidine at SQTS), and cardiac sympathetic denervation. Radiofrequency ablation (RFA) is considered to be the preferred method in idiopathic ventricular fibrillation (20%), whereas for the remaining canalopathies the frequency of RFA use does not exceed 8%.

The authors of the survey conclude that the study participants share a commitment to the present recommendations; however, they point out that more than 50% of all centers participating in the survey do not participate in any of the registers (local, national or international), which, of course, complicates the task of studying the course of the disease, effectiveness of therapy, risk stratification, and prognosis in patients with primary arrhythmogenic syndromes.

## Abbreviations

ARVD: arrhythmogenic right ventricular dysplasia
SCD: sudden cardiac death
CAD: coronary artery disease
ICD: implantable cardioverter-defibrillator
CPVT: catecholaminergic polymorphic ventricular tachycardia
LV: left ventricle
MRI: magnetic-resonance imaging
VT: ventricular tachycardia
BS: Brugada syndrome
SPR: sarcoplasmic reticulum
LV EF: left ventricular ejection fraction
VF: ventricular fibrillation
CHF: congestive heart failure
ECG: electrocardiography
PEA: pulseless electrical activity
EPS: electrophysiological study
cAMF: cyclic adenosin monophosphate
HCN-cannels: hyperpolarized activated channels
LQTS: long QT syndrome
SQTS: short QT syndrome
